# Robust classification of heart valve sound based on adaptive EMD and feature fusion

**DOI:** 10.1371/journal.pone.0276264

**Published:** 2022-12-08

**Authors:** Weibo Wang, Jin Yuan, Bingrong Wang, Yu Fang, Yongkang Zheng, Xingping Hu

**Affiliations:** 1 Department of Electrical Engineering and Electronic Information, Xihua University, Chengdu, Sichuan, China; 2 Patent Examination Cooperation Sichuan Center of the Patent Office, CNIPA, Chengdu, Sichuan, China; 3 State Grid Sichuan Electric Power Research Institute, Chengdu, Sichuan, China; 4 Sichuan Technology and Business University, Chengdu, Sichuan, China; Sant Longowal Institute of Engineering and Technology, INDIA

## Abstract

Cardiovascular disease (CVD) is considered one of the leading causes of death worldwide. In recent years, this research area has attracted researchers’ attention to investigate heart sounds to diagnose the disease. To effectively distinguish heart valve defects from normal heart sounds, adaptive empirical mode decomposition (EMD) and feature fusion techniques were used to analyze the classification of heart sounds. Based on the correlation coefficient and Root Mean Square Error (RMSE) method, adaptive EMD was proposed under the condition of screening the intrinsic mode function (IMF) components. Adaptive thresholds based on Hausdorff Distance were used to choose the IMF components used for reconstruction. The multidimensional features extracted from the reconstructed signal were ranked and selected. The features of waveform transformation, energy and heart sound signal can indicate the state of heart activity corresponding to various heart sounds. Here, a set of ordinary features were extracted from the time, frequency and nonlinear domains. To extract more compelling features and achieve better classification results, another four cardiac reserve time features were fused. The fusion features were sorted using six different feature selection algorithms. Three classifiers, random forest, decision tree, and K-nearest neighbor, were trained on open source and our databases. Compared to the previous work, our extensive experimental evaluations show that the proposed method can achieve the best results and have the highest accuracy of 99.3% (1.9% improvement in classification accuracy). The excellent results verified the robustness and effectiveness of the fusion features and proposed method.

## Introduction

Heart sound is a weak biological signal produced by the systolic and diastolic motion of the human heart. It is also a vibration signal with nonlinear and non-stationary characteristics. Rheumatic heart disease refers to the effect of rheumatic fever on the heart valve, which results in heart valve disease. Its symptoms are stenosis or insufficiency of the mitral valve, tricuspid valve, and aortic valve. With the aging of the population, senile valvular disease, coronary heart disease, and valvular disease caused by myocardial infarction are becoming more and more common [1]. For a long time, the feature extraction of heart sound signals has been a research hotspot in the biomedical field. Existing research has put forward a variety of heart sound feature extraction from the perspectives of time domain, frequency domain, and time-frequency domain. Among them, wavelet analysis is widely used because of its excellent ability to represent local signal information in time and frequency domains. Used for time-frequency feature extraction of the heart sound signals, heart sound analysis based on S transform is an extension of wavelet transform and STFT, which overcomes the deficiencies of the latter two. However, most of these methods are based on the concept of linear time-varying or time-invariant. Because of the nonlinear and non-stationary characteristics of the heart sound signal, the linear analysis method is bound to ignore some important information inside the signals [2].

Empirical Mode Decomposition (EMD) is a new method for non-stationary processing signals proposed by Huang [3], a Chinese scientist of NASA in Hilbert-Huang Transform essential part. Zhang et al. used an empirical mode decomposition technique (EMD) to remove wall components from mixed signals [4]. The new method improves the performance of effectively and objectively removing wall components from composite signals. The time-frequency analysis method based on EMD is suitable for analyzing nonlinear and non-stationary signals and for the analysis of linear and stationary signals, which can adaptively decompose any signal into multiple intrinsic mode functions (IMF). Each IMF component contains the local characteristics of the original signal in different time scales. Analysis of IMF components can more accurately reflect the relevant information of the detailed characteristics of the original signal. Therefore, using EMD to decompose the complex heart sound signal and then extracting the characteristic information of the signal from the decomposed IMF components can reflect the intrinsic essence of the heart sound.

Feature extraction and analysis of heart sound signals is a significant part of establishing a cardiovascular disease diagnosis system [5–7]. Different features can reflect the state of heart function from various aspects. Therefore, statistical analysis of heart sound signals can determine the difference between heart valve defects and normal heart sounds, which can be used to discriminate different sound signals. Therefore, the corresponding ordinary feature sets are extracted from the time domain [8], frequency domain [9] and nonlinear space [10, 11]. In addition to extracting the above features, the characteristics of four cardiac reserve times (T_1_, T_2_, T_11_ and T_12_) were also integrated in this paper [12]. Feature selection methods in machine learning play an important role in biomedical data analysis [13]. Feature selection techniques can be roughly divided into three types: filter method, embedded method, and wrapper method [14]. Filter methods can be divided into two main groups, namely single feature evaluation and subset feature evaluation [15], regardless of classifier design. Wrappers and embedded methods interact with classifiers to achieve feature selection [16].

In recent years, more and more researchers have adopted DL and ML for classification studies [17–20]. Commonly used classification algorithms contain algorithmic models such as artificial neural networks (ANN) [20], support vector machines (SVM), random forests (RF), maximum likelihood classifiers (MLC) [21–24], decision trees [25], KNN [26], etc. Haq et al. have been more studied in this area, where SMOTEDNN [27], CDLSTM [28], DNNBoT [29], etc., have played a central role in classification research. Convolutional neural network (CNN), recurrent neural network (RNN) methods, and some conventional methods developed in the last five years have been used extensively in heart sound classification [30].

This paper proposes a feature reconstruction algorithm and feature fusion method based on adaptive EMD to classify heart valve diseases. It can effectively distinguish heart valve defects from normal heart sounds by selecting an essential subset of features. The main contributions are outlined as follows.

1. This paper improves an adaptive reconstruction method based on Hausdorff distance. After EMD transformation, the Hausdorff distance (HD) between IMFs and the original heart sound signal was calculated. Then, according to the adaptive threshold based on Hausdorff distance, the appropriate IMF components were selected to reconstruct the heart sound signal. The proposed method has a better noise reduction effect, and the reconstructed heart sound signal has more obvious feature information.
2. A feature fusion method is proposed, which extracts not only the time domain, frequency domain, and nonlinear features but also fuses four cardiac reserve times features. The proposed feature fusion method can improve the effectiveness and accuracy of heart sound classification.

## Algorithm design of preprocessing

**Fig 1:**
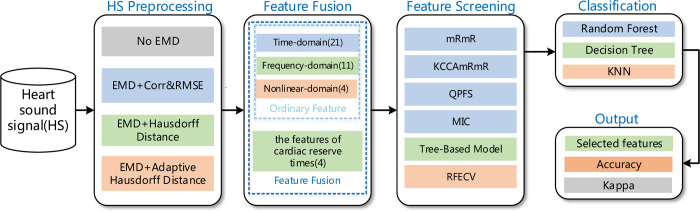
Overall method flow chart.

Fig 1 shows the overall method block diagram, which contains four main parts: preprocessing of original heart sound, feature extraction, feature screening, and classification. Heart sound is a kind of weak physiological signal, which will inevitably produce large or small noise due to various types of interference in the acquisition process. Such noise may cover up the original characteristics of the signal and affect the subsequent analysis of different heart sound signal types. Noise mainly includes environmental noise, power frequency noise, friction sound of skin contacts and collection equipment, the instrument’s interference, etc. Therefore, to maximize the retention of valuable signals, it is necessary to preprocess the original signal.

### A. EMD

The Hilbert-Huang transform includes Huang transform and Hilbert spectrum analysis. Huang transform is also called Empirical Mode Decomposition (EMD) [10, 11]. EMD, as a nonlinear and non-stationary signal analysis method, can decompose the heart sound signal into several intrinsic mode functions, and each IMF component carries local characteristics corresponding to the signal. Compared with wavelet transform, EMD decomposes the signal by selecting the wavelet function in advance and adaptively decomposes each IMF component according to frequency from high to low. Each IMFs contains the local characteristics of different time scales of the original signal. The IMF component must satisfy two restrictions: In the whole sequence, the number of extreme points *a* and zero-crossing points *b* meet the condition of |*a*-*b*| ≤ 1; For any point, the mean value of the upper and lower envelopes composed of extreme local issues must be 0.

**Fig 2:**
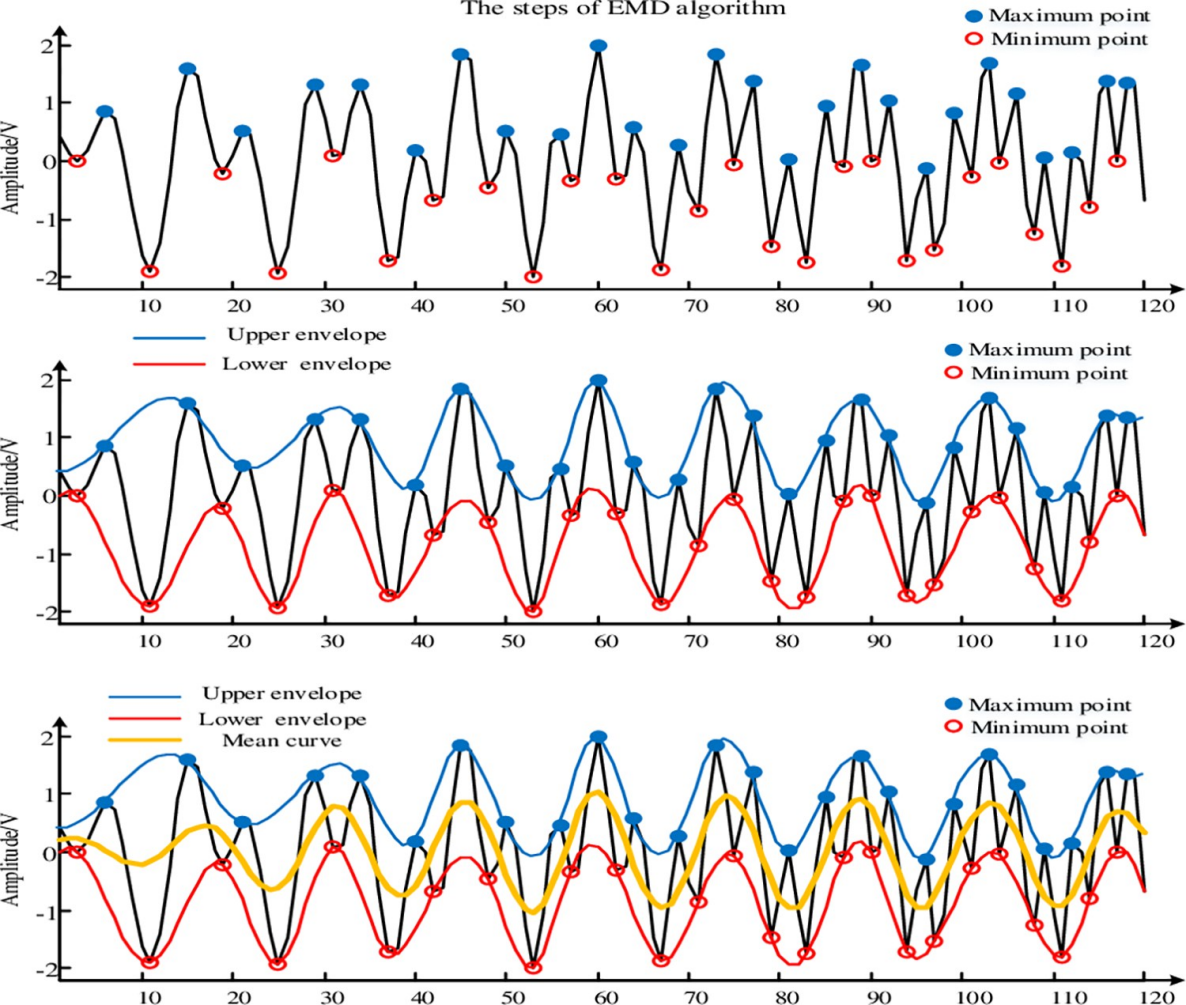
EMD decomposition principle.

For a time-series signal *s*(*t*), the principle of EMD is as follows [31]:

1. Determine the local maximum and minimum points of the original heart sound signal *s*(*t*), and fit the upper and lower envelopes *e*_1_(*t*) and *e*_2_(*t*), as shown in the yellow and green curves in Fig 2.
2. Obtain the mean curve *m*(*t*) of *e*_1_(*t*) and *e*_2_(*t*) as shown in Formula (1), as shown in the red curve in Fig 2.
$$m(t)=(e_{1}(t)+e_{2}(t))/2$$ (1)
  Calculate the mean curve *m*(*t*) of the envelopes, and uses(*t*) minus *m*(*t*) to get:
$$h_{1}(t)=s(t)-m(t)$$ (2)
  If *h*_*1*_(*t*) does not satisfy any of the above conditions of IMF, then regard *h*_1_(*t*) as *s*(*t*), and repeat the above steps until the component *h*_*k*_(*t*) that satisfies the restrictions is obtained, and record it as the first IMF component *c*_1_(*t*).
3. Calculate the component *r*_1_(*t*) = *s*(*t*)-*c*_1_(*t*), use *r*1(*t*) as the original signal, and repeat the above steps until the end of the decomposition, and finally obtain an IMF component and a remainder *r*(*t*). So far, the original signal can also be expressed as:
$$s(t)=\overset{n}{\underset{i=1}{\sum }}c_{i}(t)+r_{n}(t)$$ (3)

**Fig 3:**
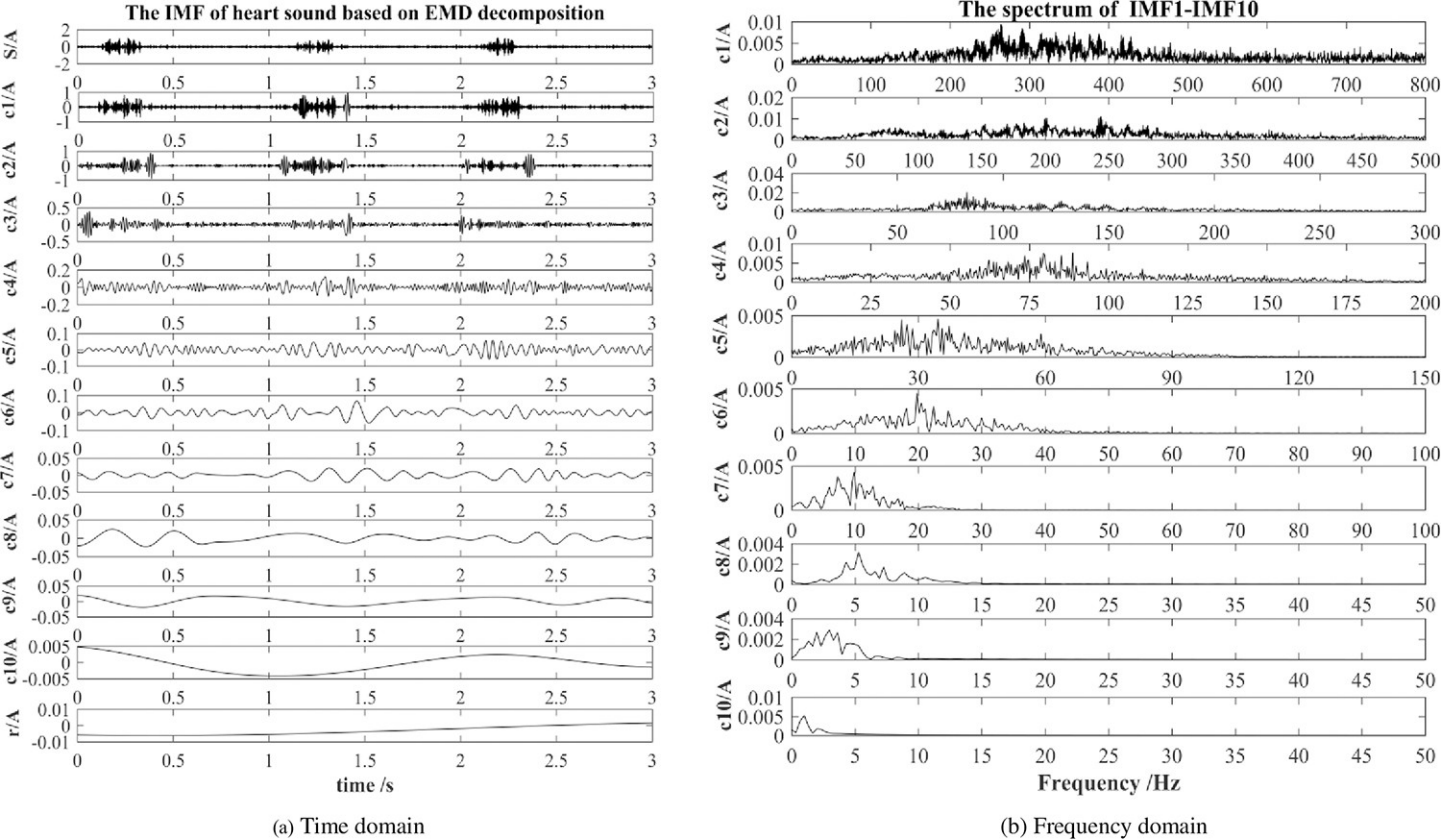
The EMD decomposition of heart sound. (a) Time domain; (b) Frequency domain.

Fig 3 shows the IMF component signal’s time and frequency domain waveforms obtained from an abnormal heart sound valve defect signal (Aortic stenosis, AS) after EMD. The number of IMF components obtained by decomposition from different signals is different. The figure only shows the first ten components (*c*_1_-*c*_10_) and the remainder *r* of the empirical mode decomposition. Observing from Fig 3(B), after EMD processing, the original heart sound signal containing multiple frequency bands is decomposed into multi-layer IMF components in ascending order of frequency. The frequency areas of the heart sound signal are mainly concentrated in the low-frequency part.

### B. IMF selection and reconstruction

Since the heart sound signal collection will inevitably generate noise due to various interferences, the original characteristics of the heart sound signal will be masked by the noise. It can be seen from Fig 3 that the essential information of the original signal is often concentrated in a few IMF components. And the interference caused by noise to the signal characteristics can be effectively reduced by screening suitable IMF component signals. We choose two evaluation indicators: Correlation Coefficient [32, 33], and Root Mean Square Error [34].

#### 1) Correlation and root mean squared error

The correlation coefficient formula based on EMD is as follows:

$$Corr_{i}=\frac{\sum_{j=1}^{N}\left(s(j)-\bar{s(j)})(c_{i}(j)-\bar{c_{i}(j)}\right)}{\sqrt{\sum_{j=1}^{N}{\left(s(j)-\bar{s(j)}\right)}^{2}\sum_{j=1}^{N}{\left(c_{i}(j)-\bar{c_{i}(j)}\right)}^{2}}}$$ (4)

In Formula (4), *corr*_*i*_ is the correlation coefficient between the original heart sound signal *s*(*j*) and the IMF component signal, *j* is the *j*-th sample of the signal, and *c*_*i*_(*j*) is the *j*-th of the *i*-th IMF component obtained by EMD. Where: *i* = 1, 2,…, *L*. The larger the *corr*, the higher the correlation.

The *RMSE* formula based on EMD is as follows:

$$RMSE_{i}=\sqrt{\frac{\sum_{j=1}^{N}{\left(s(j)-c_{i}(j)\right)}^{2}}{N}}$$ (5)

In Formula (5), *RMSE*_*i*_ is the Root Mean Square Error between the original heart sound signal *s*(*j*) and the IMF component signal, often used as an error measurement. The smaller the *RMSE* value, the higher the closeness to the original signal.

According to the *Corr*_*i*_ and *RMSE*_*i*_ of the component signal and the original signal, the threshold *λ* and *δ* are calculated by the Formula (6). The judgment condition Formula (7) is used for adaptive threshold judgment (the correlation coefficient of the IMF component is greater than or equal to *λ*, and the Root Mean Square Error is smaller than *δ*) to filter out practical IMF components. Because the *L* obtained by decomposition of each signal is different, the threshold values *λ* and *δ* are different, and the number of IMF components screened is also different.


$$\begin{array}{l} \left\{ \begin{array}{l} \lambda =\frac{\left(\sum_{i=1}^{L-1}Corr_{i}\right)}{L-1}\\ \delta =\frac{\left(\sum_{i=1}^{L-1}RMSE_{i}\right)}{L-1} \end{array}\right.\\ L:\;\mathrm{the}\;\mathrm{number}\;\mathrm{of}\;\mathrm{IMF}\;\mathrm{components} \end{array}$$ (6)

$$Corr_{i}\geq \lambda ,\&RMSE_{i}\leq \delta$$ (7)

Since each IMF component signal carries noise, the noise is not correlated with the heart sound signal and has a significant deviation. This article uses an adaptive idea to select the excellent IMF components and reconstruct them to form a sub-signal for subsequent feature extraction.

#### 2) Algorithm flow

**Fig 4:**
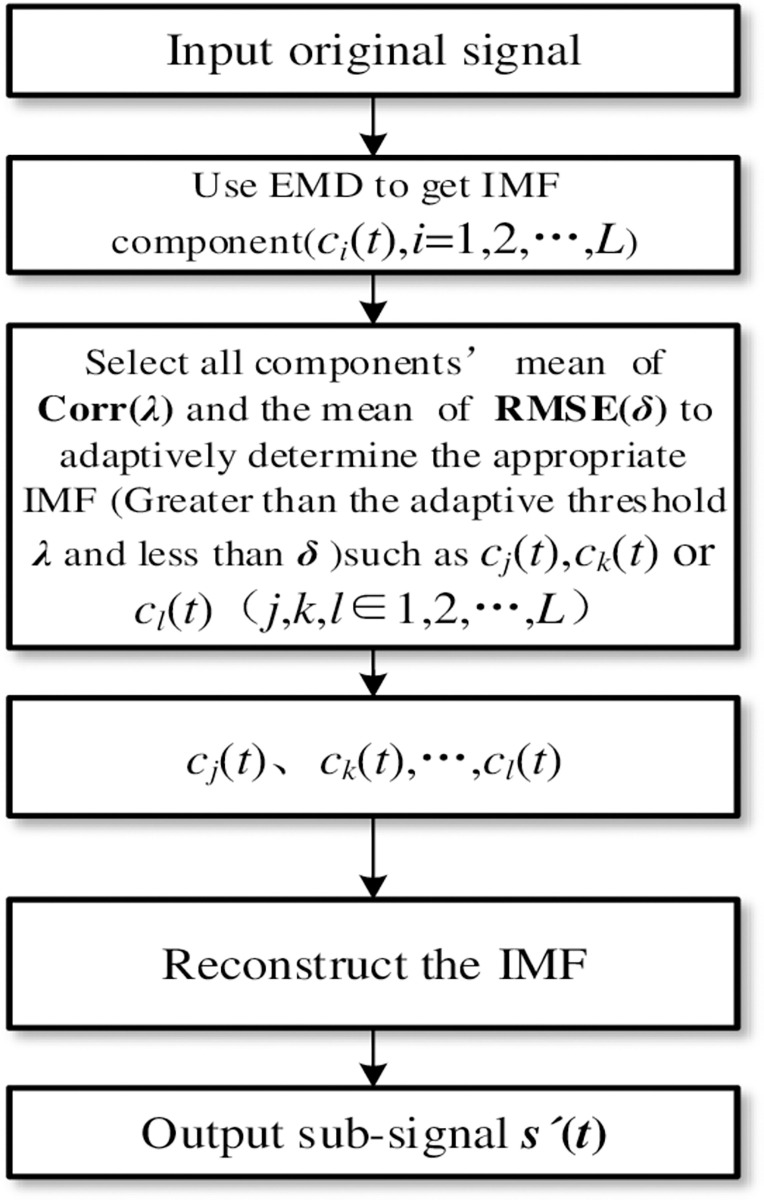
The flowchart based on EMD with adaptive reconstruction.

Fig 4 shows the adaptively reconstruction. Firstly, the Corr and RMSE between each IMFs component signal and the original heart sound signal are calculated. The best component signal is selected according to the adaptive threshold selection rule. Finally, the heart sound sub-signal is reconstructed. The signal can more intensively characterize the adequate information of the original heart sound and prepare for the extraction of various characteristic parameters of the heart sound. Fig 6(A) and 6(B) respectively show the AS, MS, MR, MVP, and NHS heart sound signals, as well as the correlation coefficient size and RMSE of the first 9-layer components after ASD, VSD, TOF, and NHS heart sounds, are decomposed. Among them, the dotted lines represent the adaptive thresholds *λ* and *δ*. The IMF components lower than *λ* and higher than *δ* are the original signal interference components, represented by black bars. The IMF components higher than *λ* and lower than *δ* are the most robust signal correlated features, represented by yellow and blue bars, respectively.

### C. Improved adaptive reconstruction based on Hausdorff distance

Based on the above adaptive reconstruction IMF method, this paper also finds that using the first seven layers of IMF components can optimize the results. So, calculate the Hausdorff Distance (HD) value of each IMF and the original signal, and then adaptively select the appropriate IMF component reconstruction [35].

#### 1) Hausdorff distance

The Hausdorff Distance (HD) is the Max-Min distance between two geometric objects to measure their degree of resemblance [8]. Given two nonempty points sets *A = {a*_*1*_, *a*_*2*_,*…*, *a*_*m*_*}* and *B = {b*_*1*_, *b*_*2*_,*…*, *b*_*n*_*}*, the HD between *A* and *B* is formulated as

H(A,B)=max[h(A,B),h(B,A)] (8)

where we have:

$$h(A,B)=\underset{a\in A}{\max}(\underset{b\in B}{\min}\|a-b\|)$$ (9)

$$h(B,A)=\underset{b\in B}{\max}(\min\|a-b\|)$$ (10)

where ||*|| is a norm distance metric, called the Euclidean distance. *H*(*A*,*B*) denotes the (bid-directional) HD in *A* with *B*, which is a fundamental norm of the HD, *h(A*, *B)* is called the directed HD from *A* to *B*, and *h(B*, *A)* is called the directed HD from *B* to *A*.

#### 2) Proposed algorithm flow

The proposed algorithm flow chart is as follows.

**Fig 5:**
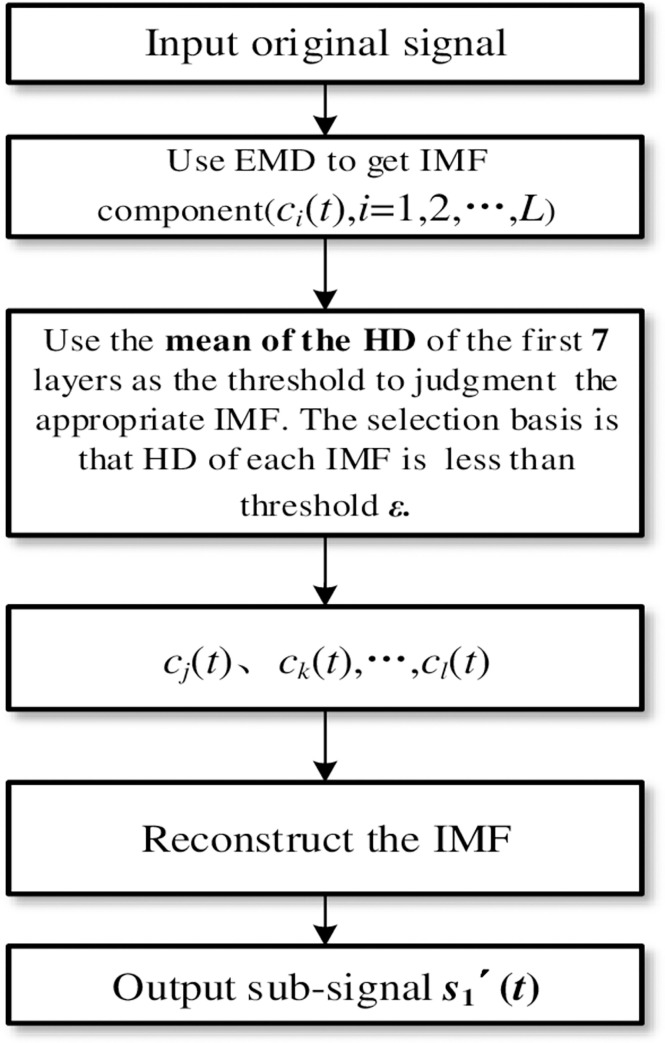
Heart sound flowchart based on EMD adaptive HD reconstruction.


As shown in Fig 5, this paper improves the method of Hausdorff adaptive threshold to IMF and further selects more appropriate IMF components, as shown in the following

$$\varepsilon ==\frac{\left(\sum_{i=1}^{L-1}HD_{i}\right)}{7}$$ (11)

where L is the number of IMF components.

Fig 5 shows the improved adaptive threshold selection method based on HD. First, after obtaining all the IMF components after EMD, calculate the HD between each layer and the original signal, and then sort the IMF components with the HD value in the first seven layers. Therefore, the appropriate IMF component can be adaptively selected by calculating the mean of the IMF components’ HD from the first seven layers. Finally, the heart sound sub-signal is reconstructed.

**Fig 6:**
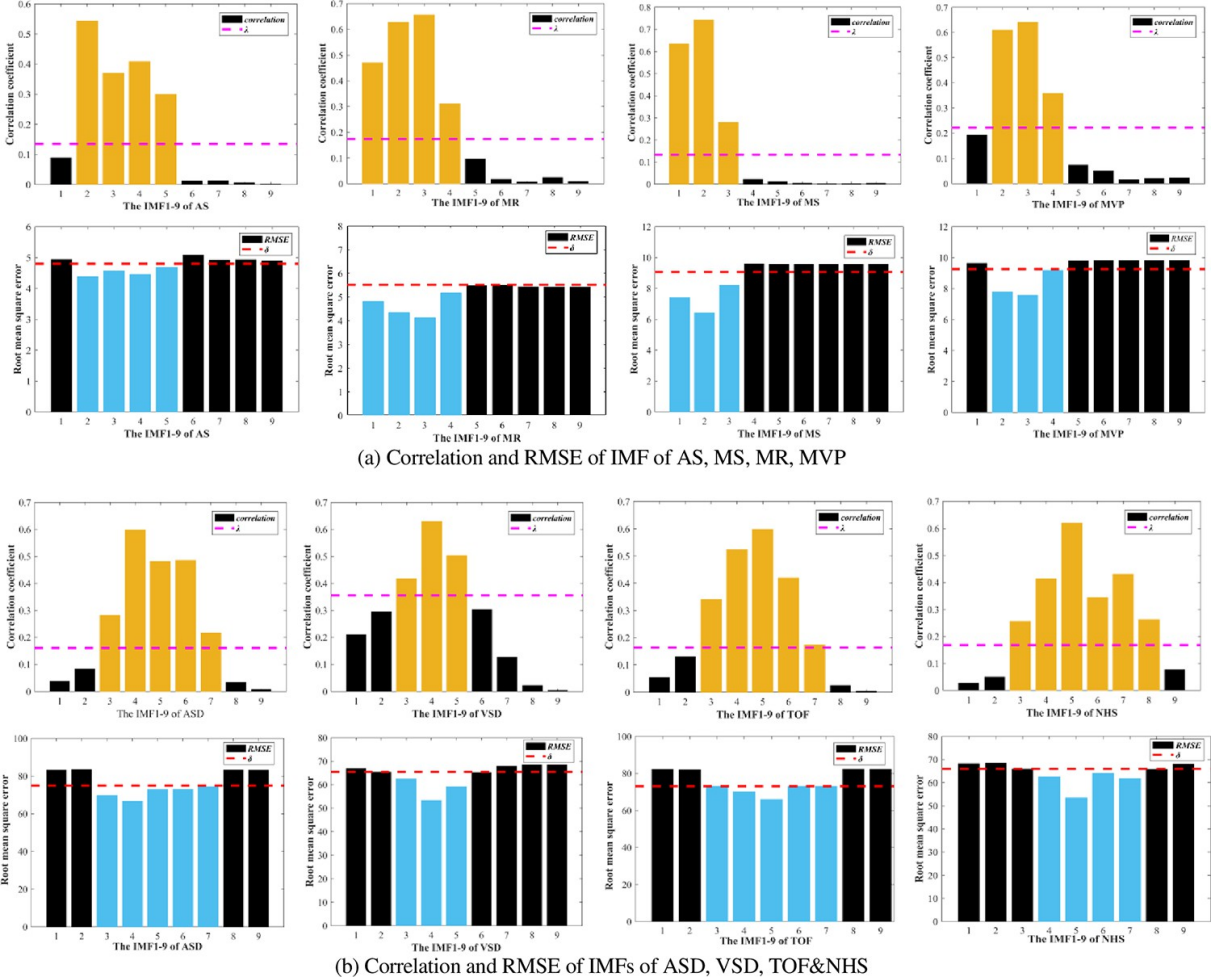
Correlation and RMSE of IMFs of heart sound. (a) Correlation and RMSE of IMF of AS, MS, MR, MVP; (b) Correlation and RMSE of IMFs of ASD, VSD, TOF&NHS.

**Fig 7:**
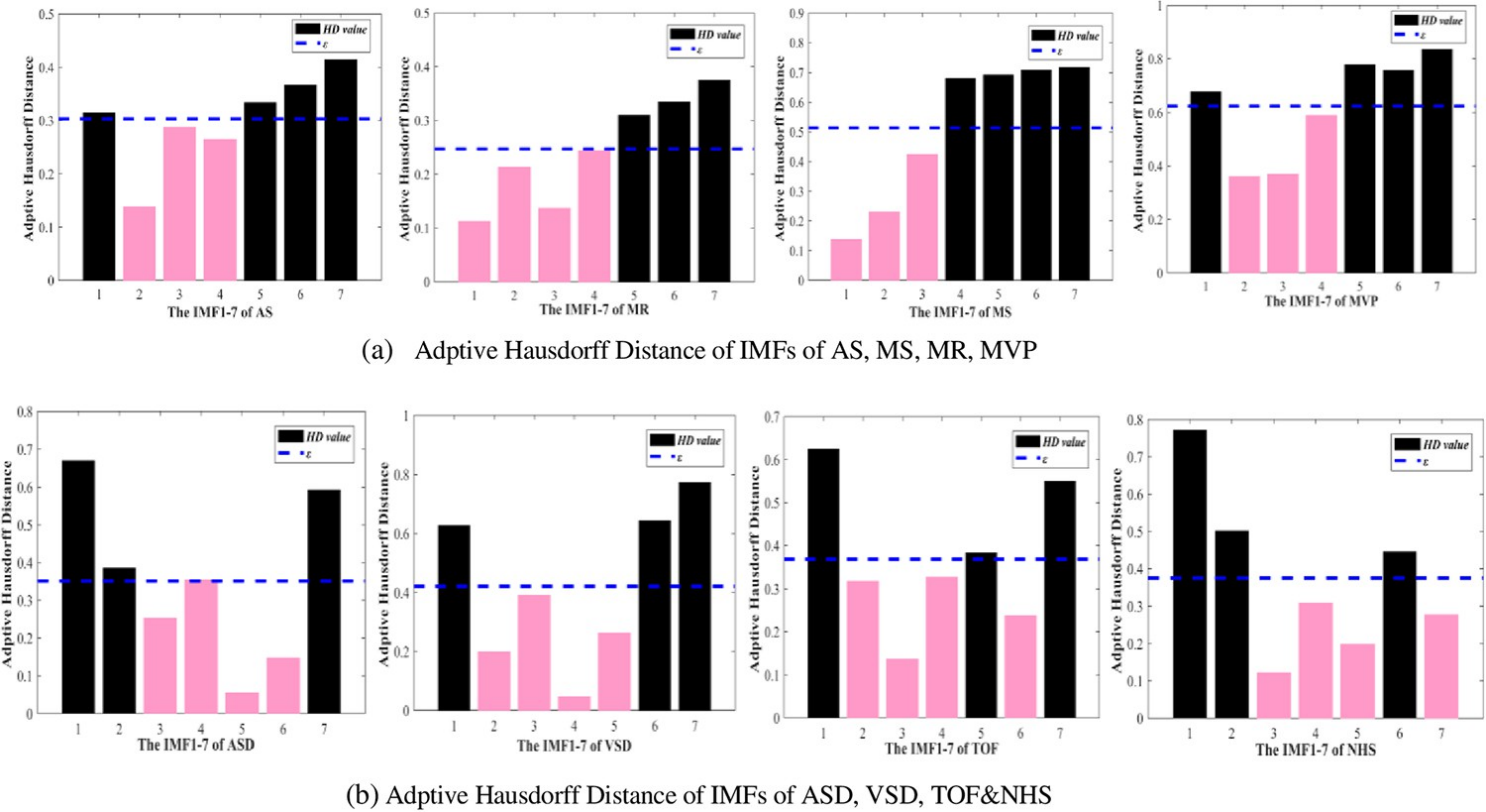
Adaptive selection based on the mean of the first 7 layers of HD. (a) Adptive Hausdorff Distance of IMFs of AS, MS, MR, MVP; (b) Adptive Hausdorff Distance of IMFs of ASD, VSD, TOF&NHS.

Figs 6 and 7 show the comparison results using the two IMF components selection method. In Fig 6(A), according to Formula (7), Corr&RMSE threshold selection method is applied to the abnormal heart sound AS to select 2th-5th layers of IMF to reconstruct the signal. In Fig 7(A), according to Fig 5, the AS’ IMF components 2th-5th layers smaller than the threshold of adaptive Hausdorff Distance *ε* are selected.

**Fig 8:**
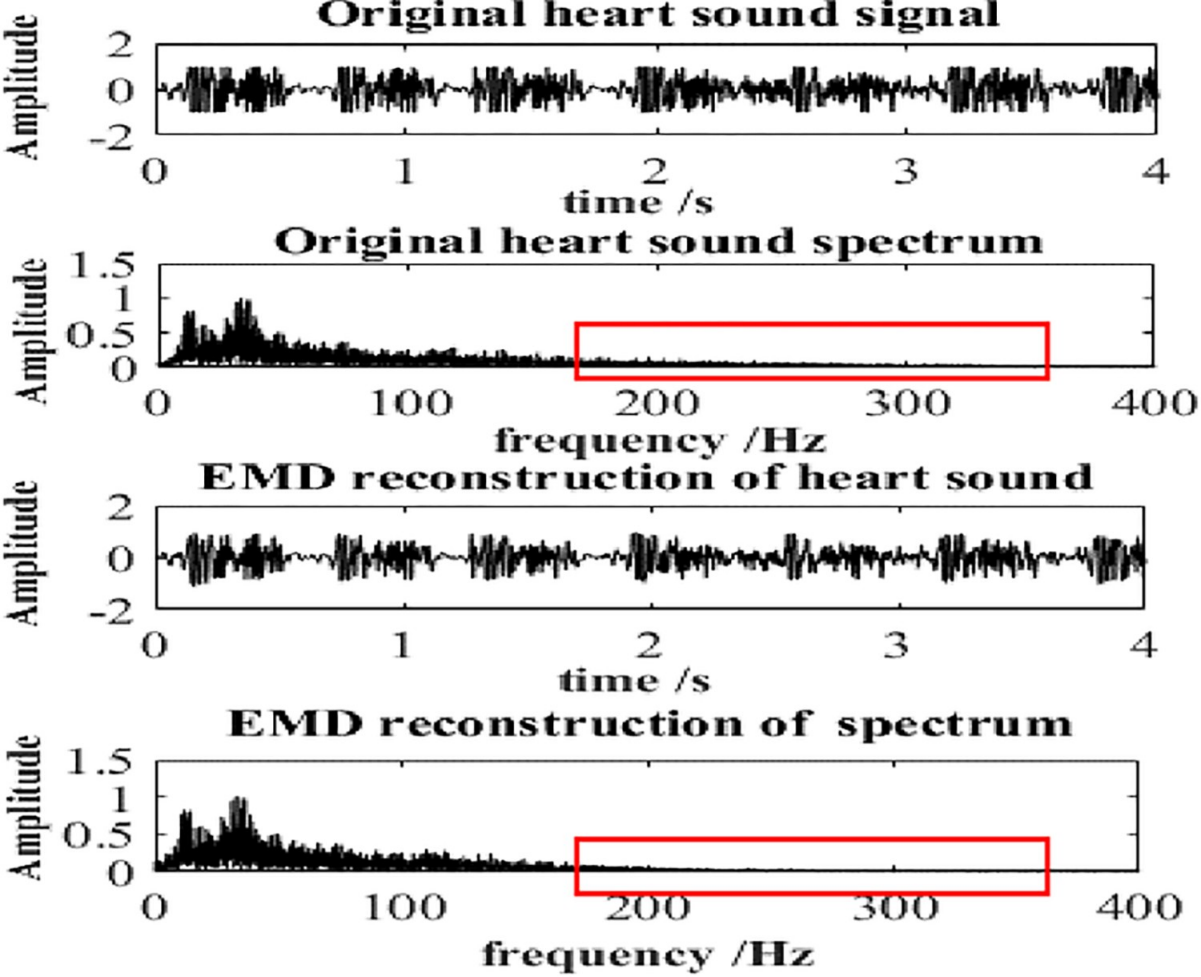
Time-frequency of heart sound EMD reconstruction.

Fig 8 is a time-frequency waveform of the heart sound signal before and after the EMD adaptive filtering and reconstruction. It can be seen from the red box marked that the noise in the high-frequency part of the heart sound is effectively suppressed due to the reconstruction. Therefore, the reconstruction operation can reduce the noise of the heart sound signal effectively, thus can successfully select heart sound sub-signals that are more similar to the original heart sound.

## Feature fusion and selection

### A. Feature fusion

Different features can reflect the state of heart function from various aspects. Therefore, statistical analysis of heart sound signals can obtain the difference between heart valve defect sounds and standard heart sound signals, which can be used to discriminate different heart sound signals. Existing studies have shown that the waveform transformation characteristics, energy characteristics and complexity characteristics of heart sound signals can reflect the corresponding cardiac activity state of different heart sound samples. Therefore, this section extracts complementary feature sets from the time domain, frequency domain and nonlinear domain and combines four cardiac reserve time features. Then the analysis can be used for subsequent feature screening and classification recognition.

#### 1) Time domain features

This paper is from the perspectives of mathematical statistics, vibration signal feature analysis, and heart sound time-domain waveforms. There are 25 kinds of time-domain features, including Mean, Mean Square, Maximum, Minimum, Variance, Standard Deviation, Root Mean Square (RMS), Peak-to-Peak Value, Root-Square Amplitude, Skewness Factor, Kurtosis Factor, Form Factor, Crest Factor, Impulse Factor, Margin Degree Factor, Hjorth Parameter (Mobility), Hjorth Parameter (Complexity), First Quartile, Second Quartile, Third Quartile, Interquartile Range.

#### 2) Frequency domain features

In addition to time-domain signal analysis, the Fourier transform can also be used to observe the frequency domain characteristics of signals that cannot be obtained in the time domain.

This paper uses analysis methods such as DFT and Hilbert transform to extract 11 features from the frequency perspective, including Energy Entropy, Shannon Entropy, Mean Frequency, Center of Gravity Frequency, Root Mean Square Frequency, and Frequency Deviation, Instantaneous Energy Median, Average Instantaneous Energy, Median Instantaneous Frequency, Average Instantaneous Frequency.

#### 3) Nonlinear domain features

This kind of information describes the state of movement and the existence of objective things. Information entropy illustrates the complexity of data from the perspective of information theory. It also expands and analyzes from the standpoint of nonlinearity to obtain corresponding load-type indicators that describe signal data. From the perspective of nonlinearity, the corresponding load-type index describing the signal data can be obtained. There are four kinds of nonlinear characteristics: approximate entropy [36], Sample Entropy, Multi-Scale Permutation Entropy, and Exponential Entropy.

As shown in Table 1 below, the feature set extracted in our paper includes the above 25 types of time-domain features, 11 types of frequency-domain features, and four types of nonlinear characteristics. After feature extraction, 40 types of features are labelled to facilitate subsequent feature screening experiments.

**Table 1:** 40 fusion features.

No.	Feature Name	Formula or Explanation	No.	Feature Name	Formula or Explanation
**1**	**T**_**1**_	S_1_ time width	**21**	Complexity	
**2**	T_2_	S_2_ time width	**22**	First Quartile(Q_1_)	The 25th percentile of the data in ascending order
**3**	T_11_	The cardiac reserve time from the previous S_1_ to the next S_1_	**23**	Second Quartile (Q_2_)	The 50th percentile of the data in ascending order
**4**	T_12_	The cardiac reserve time from S_1_ to S_2_ in a cycle	**24**	Third Quartile (Q_3_)	The 75th percentile of the data in ascending order
**5**	Mean	$\mu =E(X)=\underset{N\to \infty }{\lim}\frac{1}{N}\sum_{i=1}^{N}X_{i}$	**25**	Interquartile Range(IQR)	
**6**	Mean Square	$MSV=\varphi_{x}^{2}=\underset{N\to \infty }{\lim}\frac{1}{N}\sum_{i=1}^{N}X_{i}^{2}=E[X^{2}]$	**26**	Energy Entropy	
**7**	max	Maximum amplitude in the signal	**27**	Sample Entropy	
**8**	min	Minimum amplitude in the signal	**28**	Approximate Entropy	
**9**	Variance	$\sigma_{X}^{2}(t)=\underset{N\to \infty }{\lim}\frac{1}{N}\sum_{i=1}^{N}{\left[X_{i}(t)-\mu_{x}(t)\right]}^{2}$	**29**	Shannon Entropy		

**10**	Standard Deviation	$\sigma_{X}(t)=\sqrt{\underset{N\to \infty }{\lim}\frac{1}{N}\sum_{i=1}^{N}{\left[X_{i}(t)-\mu_{x}(t)\right]}^{2}}$	**30**	Multi-Scale Permutation Entropy	
**11**	Root Mean Square	$X_{RMS}=\sqrt{\frac{\sum_{i=1}^{N}{\left(X_{i}\right)}^{2}}{N}}$ $X_{\mathrm{peak}}=\max(X)-\min(X)$	**31**	Exponential Entropy	
**12**	Peak-to-Peak Value		**32**	Mean Square Frequency	
**13**	Root-Square Amplitude	$X_{RM}={\left[\frac{1}{N}\sum_{i=1}^{N}\sqrt{\left|x_{i}\right|}\right]}^{2}$	**33**	Center of Gravity Frequency	
**14**	Skewness	$\mathrm{Skew}=E[{\left(\frac{X-\mu }{\sigma }\right)}^{3}]=\frac{\mu_{3}}{\sigma^{3}}$	**34**	Root Mean Square Frequency	
**15**	Kurtosis	$\mathrm{kurt}(X)=E[{\left(\frac{X-\mu }{\sigma }\right)}^{4}]=\frac{\mu_{4}}{\sigma^{4}}$	**35**	Frequency Standard Deviation		

**16**	Crest	$S_{X}(t)=\frac{\sqrt{\underset{N\to \infty }{\lim}\frac{1}{N}\sum_{i=1}^{N}X_{i}^{2}(t)}}{\underset{N\to \infty }{\lim}\frac{1}{N}\sum_{i=1}^{N}|X_{i}|}=\frac{\sqrt{E[X^{2}(t)]}}{E[|X|]}$	**36**	Instantaneous Energy Max-min Deviation	——
**17**	Crest	$C=\frac{X_{\mathrm{peak}}}{X_{RMS}}$	**37**	Instantaneous Energy Median	——
**18**	Impulse	$X_{IMP}=\frac{X_{\mathrm{peak}}}{\left[\frac{1}{N}\sum_{i=1}^{N}|x_{i}|\right]}$ $X_{L}=\frac{X_{\mathrm{peak}}}{{\left[\frac{1}{N}\sum_{i=1}^{N}\sqrt{\left|x_{i}\right|}\right]}^{2}}$	**38**	Average Instantaneous Energy	——
**19**	Margin Degree		**39**	Median Instantaneous Frequency	
**20**	Mobility	$H_{M}=\frac{\sigma_{1}}{\sigma }$	**40**	Average Instantaneous Frequency	

#### 4) Cardiac reserve time features

According to the established single-degree-of-freedom vibration model, auscultation of heart sound is compared to the duration of low-frequency sound or sound pressure captured by the tympanic membrane to extract the corresponding time-limited features. T_1_ is the time limit of S_1_, T_2_ is the time limit of S_2_, T_12_ is the time limit of S_1_ to S_2_ in the same cardiac cycle, and T_11_ is the time limit of one cardiac cycle, indicating the time interval from S_1_ to the start of the next adjacent cardiac cycle S_1_.

### B. Feature selection

The multiple features may include related, irrelevant, and redundant features. Therefore, selecting features that are beneficial to learning classification from all features is necessary. Feature selection methods can be divided into three types:

- Filter: Filtering method, scoring each feature according to divergence or correlation, setting threshold or the number of thresholds to be selected, and selecting features.
- Wrapper: according to the objective function (usually the prediction effect score), select several features at a time, or exclude several features.
- Embedded: The embedding method first uses machine learning algorithms and models for training, gets each feature’s weight coefficient and selects the feature from large to small according to the coefficient. Similar to the Filter method, but through training to determine the pros and cons of features.

The significant goals of feature selection are increasing the accuracy, finding the minimal effective feature subset, and increasing the performance of evaluations. So, this paper selects six feature screening and sorting algorithms [37], among which the filter methods include mRMR, KCCAmRMR, MIC, and QPFS, the wrapper method is RFECV, and the embedded method has a tree-based model.

#### 1) mRMR

The Minimum Redundancy-Maximum Relevance (mRMR) algorithm is a filtering feature selection method [38, 39]. This method can balance correlation and redundancy in different ways and uses mutual information as a calculation criterion to measure the redundancy between features and the relationship between characteristics and class variables. The correlation between features is selected by maximizing the correlation between characteristics and class variables and minimizing the redundancy between features.

#### 2) KCCAmRMR

An improved algorithm, called Kernel Canonical Correlation Analysis based on mRMR (KCCAmRMR) [40], was also developed, in which irrelevant redundancy is filtered out by using an additional kernel canonical correlation analysis. Thus, only the relevant redundancy is considered in the subsequent mRMR procedure. The feature selection criterion of our KCCAmRMR method has two terms as in mRMR: relevance and redundancy.

#### 3) QPFS

Quadratic programming feature selection (QPFS) is a feature ranking algorithm that uses the information theory as the similarity measure [41]. Also, it applies an optimization solution to estimate the quality of a given dataset’s features. The QPFS assigns a weight to each feature such that the more critical features will have more significant values. As a final result, the features are sorted based on decreasing consequences. Then by applying a threshold value, the top features will be selected as the last selected features.

#### 4) MIC

MIC can quantify the correlation between continuous and qualitative variables and calculate the correlation between a constant feature and qualitative target variables [42, 43]. The larger the MIC value, the stronger the recognition ability of the corresponding element. This algorithm calculates the correlation between each dimensional feature and the heart sound sample label, and essential features can be selected.

#### 5) Tree-based model

Tree-based prediction model can be used to calculate the importance of features, so they can be used to remove irrelevant features.

#### 6) RFECV

The RFECV method is divided into two parts [44]. Recursive feature elimination is used to evaluate the importance of features. The other is Cross-Validation (CV), which selects the best number of segments through CV after feature evaluation. Feature.

## Experimental results and analysis

### A. Data source

The heart sound data used in our experiment is from open source ([Online] Available: https://github.com/yaseen21khan/) [25] and data collected in our laboratory. Heart sound signals from heart valve defects were collected clinically. All recordings have been resampled to 2205Hz. The open source samples include Aortic stenosis (AS), Mitral regurgitation (MR), Mitral stenosis (MS), Mitral valve prolapse (MVP), and normal heart sounds (NHS), a total of 1000 samples. The laboratory data consists of 412 samples including ASD, VSD, TOF, and NHS. Tables 2 and 3 show the data source. The dataset was split into training data (80%) and testing data (20%).

**Table 2:** Types of samples in open source [25].

Type	AS	MS	MR	MVP	NHS	Total
Number	200	200	200	200	200	1000

**Table 3:** Types of samples in our laboratory.

Type	ASD	VSD	TOF	NHS	Total
Number	68	144	76	124	412

### B. Feature screening model comparison

This paper extracts 40 features from the heart sound reconstructed by adaptive EMD using fusion features from time, frequency, nonlinear domain and cardiac reserve time features. Each type of heart sound signal contains a 40 fusion feature data set. The open source dataset includes five types of heart sounds. To validate the proposed feature fusion technique under various EMD methods, feature sets with or without the four cardiac reserve time features were tested. Six feature screening methods are used to rank the particular importance. This performs feature reduction to prepare for the subsequent input of the classifier.

Similarly, the 412 samples collected in the laboratory were subjected to the same experiment to build the 412×40 feature set. After removing the four cardiac reserve time features, the size of the remain feature set is 412×36.

### C. Classification accuracy

**Fig 9:**
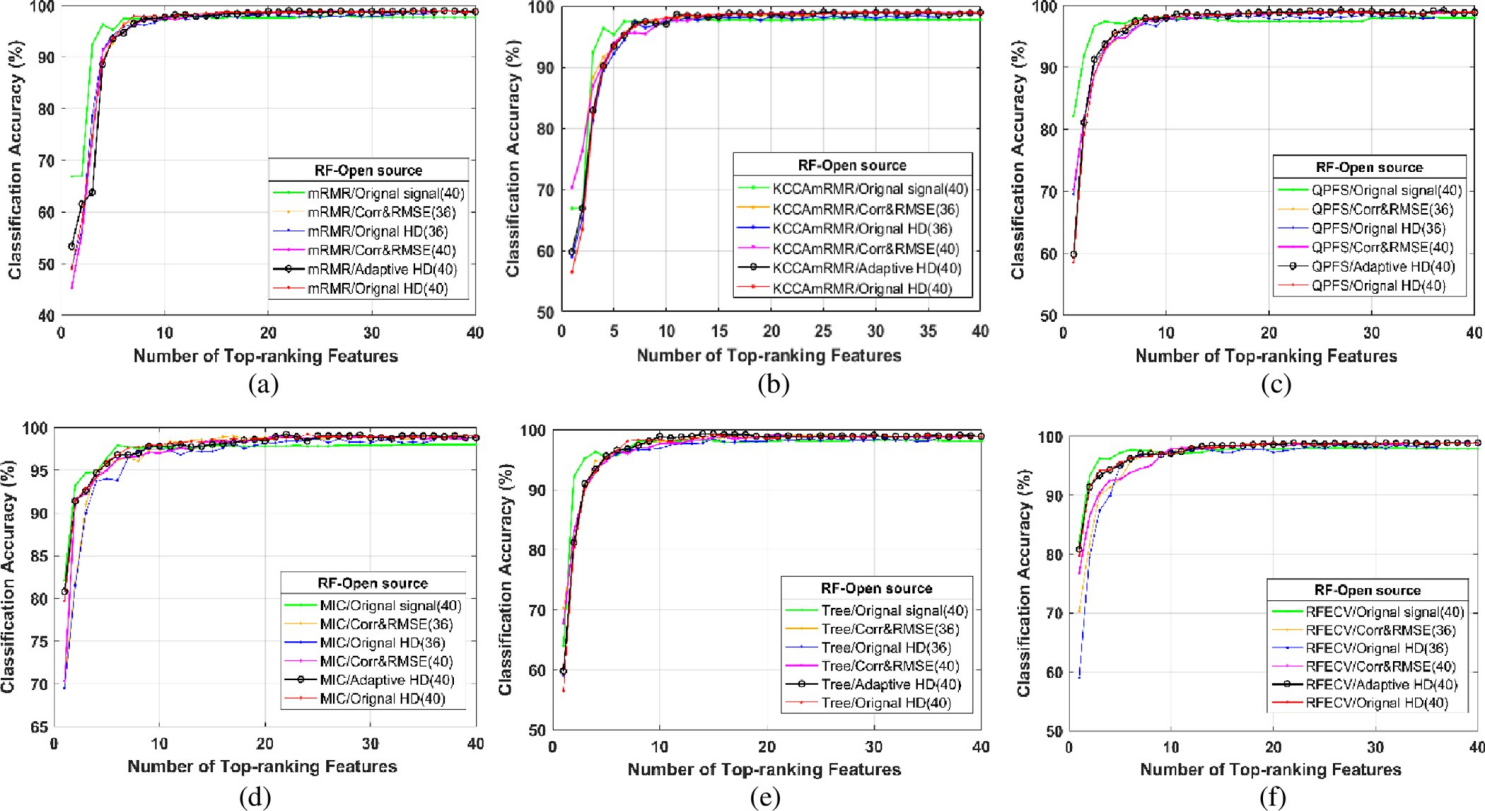
The average classification accuracy versus the number of top-ranking features selected by mRMR, KCCAmRMR, QPFS, MIC and Tree and RFECV using RF classifier from open source data.

**Fig 10:**
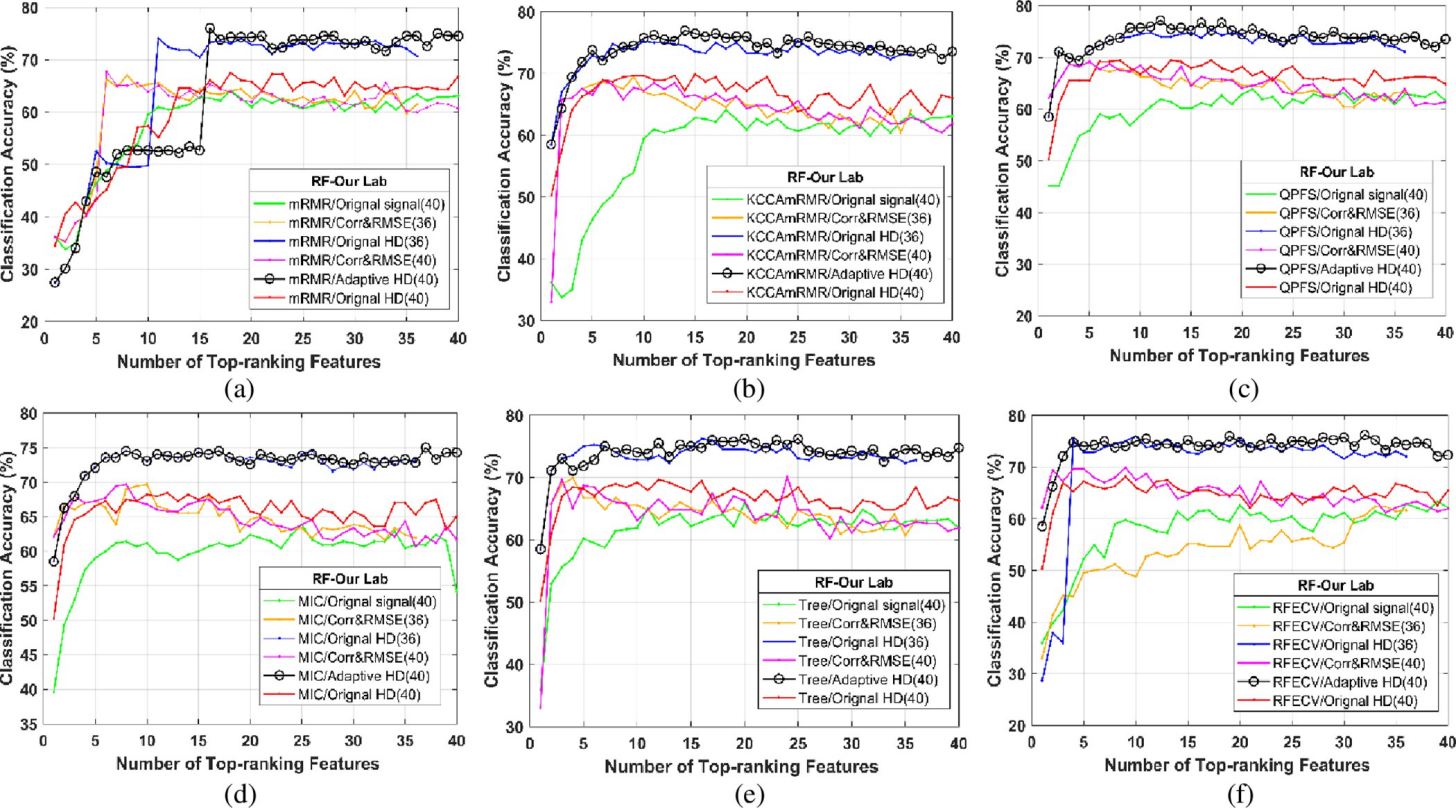
The average classification accuracy versus the number of top-ranking features selected by mRMR, KCCAmRMR, QPFS, MIC and Tree and RFECV using RF classifier from our Lab data.

From the heart sound signal in the open source database and our lab, 36/40 features ranked by mRMR, KCCAmRMR, QPFS, MIC, Tree, and RFECV were incrementally fed into the RF classifier [45, 46]. For example, the curves of the average classification accuracies versus the number of top-ranking features with open source datasets and our lab’s datasets based on 10-fold cross-validation are shown in Figs 9(A)–9(F) and 10(A)–10(F).

In Figs 9 and 10, each graph is composed of six polylines, representing a signal composition method. The composition method is as follows:

1. The original heart sound is directly extracted without the EMD reconstruction process.
2. Use Corr & RMSE to select EMD reconstructed IMF components and then extract 36 features.
3. Use the original HD to select the EMD reconstructed IMF component and perform 36 feature extraction.
4. Use Corr & RMSE to select EMD reconstructed IMF components and extract 40 features.
5. After EMD, the HD threshold of the first seven layers of IMF components is adaptively selected and reconstructed, and then extract 40 features.
6. Use the original HD to select and reconstruct the IMF components. Then, 36 feature extractions are performed. The six feature screening and sorting algorithms are used. Finally, input into the classifier.

The comparison of the six feature selection methods used in the classification experiment verifies the data mining effect of the feature data set after combining the six feature extraction data. The main idea in this experimental part is: First, select heart sound preprocessing and feature reconstruction by different methods. Secondly, 36 features and 40 features are used to compare the data set in the absence of 4 heart sound signal-specific cardiac reserve time features under the six feature screening algorithms of mRMR, KCCAmRMR, QPFS, MIC, tree-based model, and RFECV. Multi-dimensional elements are sorted to obtain a data set in which the information features of each data set are sorted from high to low. Finally, the highest-ranked feature is gradually input into the classifier to obtain the classification results.

Fig 9 shows the average classification accuracy of open source data. Under different feature selection algorithms and preprocessing methods, the corresponding average classification accuracy rises with the increase of feature dimension. After ten dimensions, the classification accuracy of each method flattens out. It can be seen from the Fig that adaptive HD performs well in all six feature screening methods.

Fig 10 shows the average classification accuracy of our laboratory data. The collected data has more noise interference than the open source data. It can be observed that, among the six signal reconstruction methods, adaptive HD has obviously higher classification accuracy than other reconstruction methods among all six feature selection methods. By comparing the blue curve and the black curve, it can find that the peak of the average accuracy curve from the extracted 36 features (without T_1_, T_2_, T_11_, and T_12_ cardiac reserve time features) is lower than that of the extracted 40 features (including T_1_, T_2_, T_11_, and T_12_). Therefore, the results of the feature fusion are better than that of the ordinary features.

Tables 4–9 compare the six different methods with six feature selection algorithms on the open-source and our laboratory datasets. The feature fusion technique is also tested by comparing 36 features (without T_1_, T_2_, T_11_, and T_12_ cardiac reserve time features) and 40 features (including T_1_, T_2_, T_11_, and T_12_). As shown in Tables 4–9, the method, No EMD, gets the worst results. And our proposed method, EMD + Adaptive HD, with 40 fusion features, can achieve the best and most robust results on the two datasets. For the open source database, the feature selection method Tree with the proposed adaptive HD can obtain the best results. The classification accuracy reached 99.3% in the open source database, and the selected features were reduced from 40 to 14 in Table 8. In the laboratory dataset, the classification accuracy reached 76.21%, and the selected features decreased from 40 to 12 in Table 6.

**Table 4:** Comparison of accuracy and feature dimension under different methods based on mRMR.

Heart Sound Dataset	Methods	A	mRMR			
			B	ACC(%)	Kappa(%)	Number of Correct &Error
Open Source	No EMD	40	11	98.00	97.50	980 & 20
	EMD + Corr&RMSE	36	28	98.80	98.50	988 & 12
	EMD + HD	36	33	98.50	98.12	985 & 15
	EMD + Corr&RMSE	40	26	99.00	98.75	990 & 10
	**EMD + Adaptive HD**	40	22	**99.00**	**98.75**	990 & 10
	EMD + HD	40	22	98.70.	98.37	987 & 13
Laboratory	No EMD	40	15	62.86	46.56	259 & 153
	EMD + Corr&RMSE	36	8	66.99	53.07	276 & 136
	EMD + HD	36	19	74.03	63.40	305 & 107
	EMD + Corr&RMSE	40	6	67.72	54.14	279 & 133
	**EMD + Adaptive HD**	40	16	**75.97**	**66.00**	313 & 99
	EMD + HD	40	18	67.48	53.84	278 & 134

The bold numbers indicate the min number of selected features or the highest accuracy. A: Number of original features; B: Number of selected features.

**Table 5:** Comparison of accuracy and feature dimension under different methods based on KCCAmRMR.

Heart Sound Dataset	Methods	A	KCCAmRmR			
			B	ACC(%)	Kappa(%)	Number of Correct &Error
Open Source	No EMD	40	30	97.90	97.37	979 & 21
	EMD + Corr & RMSE	36	26	99.00	98.75	990 & 10
	EMD + HD	36	34	98.40	98.00	984 & 16
	EMD + Corr & RMSE	40	30	99.00	98.75	990 & 10
	**EMD + Adaptive HD**	40	25	**99.00**	**98.75**	990 & 10
	EMD + HD	40	28	99.00	98.75	990 & 10
Laboratory	No EMD	40	22	65.05	49.66	268 & 144
	EMD + Corr & RMSE	36	9	69.42	56.72	286 & 126
	EMD + HD	36	10	75.24	64.91	310 & 102
	EMD + Corr & RMSE	40	6	68.93	56.06	284 & 128
	**EMD + Adaptive HD**	40	14	**76.94**	**67.30**	317 & 95
	EMD + HD	40	15	69.90	57.39	288 & 124

The bold numbers indicate the min number of selected features or the highest accuracy. A: Number of original features; B: Number of selected features.

**Table 6:** Comparison of accuracy and feature dimension under different methods based on QPFS.

Heart Sound Dataset	Methods	A	QPFS			
			B	ACC(%)	Kappa(%)	Number of Correct &Error
Open Source	No EMD	40	35	98.20	97.75	982 & 12
	EMD + Corr & RMSE	36	24	99.10	98.87	991 & 9
	EMD + HD	36	17	98.30	97.87	983 & 17
	EMD + Corr & RMSE	40	27	99.10	98.87	991 & 9
	**EMD + Adaptive HD**	40	27	**99.20**	**99.00**	992 & 8
	EMD + HD	40	25	99.00	98.75	990 & 10
Laboratory	No EMD	40	21	63.83	47.93	263 & 149
	EMD + Corr & RMSE	36	5	69.17	56.41	285 & 127
	EMD + HD	36	17	75.24	65.13	310 & 102
	EMD + Corr & RMSE	40	5	69.17	56.41	285 & 127
	**EMD + Adaptive HD**	40	12	**77.18**	**67.87**	318 & 94
	EMD + HD	40	8	69.42	57.01	286 & 126

The bold numbers indicate the min number of selected features or the highest accuracy. A: Number of original features; B: Number of selected features.

**Table 7:** Comparison of accuracy and feature dimension under different methods based on MIC.

Heart Sound Dataset	Methods	A	MIC			
			B	ACC(%)	Kappa(%)	Number of Correct &Error
Open Source	No EMD	40	33	98.00	97.50	980 & 20
	EMD + Corr & RMSE	36	17	99.00	98.75	990 &10
	EMD + HD	36	25	98.70	98.37	987 &13
	EMD + Corr & RMSE	40	22	99.10	98.87	991 & 9
	**EMD + Adaptive HD**	40	22	**99.20**	98.87	991 & 9
	EMD + HD	40	24	99.10	**99.00**	992 & 8
Laboratory	No EMD	40	20	62.38	46.06	257 & 155
	EMD + Corr & RMSE	36	10	69.66	57.39	287 & 125
	EMD + HD	36	17	74.51	64.13	307 & 105
	EMD + Corr & RMSE	40	8	69.66	57.39	287 & 125
	**EMD + Adaptive HD**	40	17	**74.51**	**64.13**	307 & 105
	EMD + HD	40	12	68.45	55.51	282 & 130

The bold numbers indicate the min number of selected features or the highest accuracy. A: Number of original features; B: Number of selected features.

**Table 8:** Comparison of accuracy and feature dimension under different methods based on tree.

Heart Sound Dataset	Methods	A	Tree			
			B	ACC(%)	Kappa(%)	Number of Correct &Error
Open Source	No EMD	40	22	98.30	97.87	983 & 17
	EMD + Corr & RMSE	36	21	99.20	99.00	992 & 8
	EMD + HD	36	20	98.10	97.62	981 & 19
	EMD + Corr & RMSE	40	21	99.20	99.00	992 & 8
	**EMD + Adaptive HD**	40	14	**99.30**	**99.12**	993 & 7
	EMD + HD	40	15	99.20	99.00	992 & 8
Laboratory	No EMD	40	20	65.78	51.02	271 & 141
	EMD + Corr & RMSE	36	4	69.90	57.73	288 & 124
	EMD + HD	36	16	76.21	66.43	314 & 98
	EMD + Corr & RMSE	40	24	70.15	58.05	289 & 123
	**EMD + Adaptive HD**	40	20	**76.21**	**66.43**	314 & 98
	EMD + HD	40	12	69.66	57.18	287 & 125

The bold numbers indicate the min number of selected features or the highest accuracy. A: Number of original features; B: Number of selected features.

**Table 9:** Comparison of accuracy and feature dimension under different methods based on RFECV.

Heart Sound Dataset	Methods	A	RFECV			
			B	ACC(%)	Kappa(%)	Number of Correct &Error
Open Source	No EMD	40	23	98.00	97.50	980 & 20
	EMD + Corr & RMSE	36	21	98.90	98.62	989 & 11
	EMD + HD	36	26	98.40	98.00	984 & 16
	EMD + Corr & RMSE	40	27	98.90	98.62	989 & 11
	**EMD + Adaptive HD**	40	22	**98.90**	**98.62**	989 & 11
	EMD + HD	40	22	98.90	98.62	989 & 11
Laboratory	No EMD	40	36	62.86	46.15	257 & 155
	EMD + Corr & RMSE	36	33	62.38	46.66	259 & 153
	EMD + HD	36	10	75.97	66.13	313 & 99
	EMD + Corr & RMSE	40	9	69.90	57.45	288 & 124
	**EMD + Adaptive HD**	40	32	**76.21**	**66.29**	314 & 98
	EMD + HD	40	9	68.20	55.20	281 & 131

The bold numbers indicate the min number of selected features or the highest accuracy. A: Number of original features; B: Number of selected features.

The computer used to run the proposed method is Legion R9000P2021H, and the processor is AMD Ryzen 7. The operating system is Windows 10. The time consumption of classification of heart valve sound for every sample in the open source dataset using EMD+Adaptive HD is about 10s.

Table 10 shows the classification results using different classifiers (RF, KNN, DT) on the open-source and our laboratory databases. Forty fusion features are extracted for each dataset. It can be seen that RF can achieve the best results in both datasets. The heart sound classification accuracy on the open source database reaches 99.3% when the 14 features are selected. The heart sound classification accuracy on the laboratory database was 77.18% when the 12 features were selected.

**Table 10:** The best accuracy and number of selected minimum features by adaptive HD method.

Heart Sound Dataset	Number of Samples	A	RF	KNN	DT
Open Source	1000	40	B/ACC(%)	B/ACC(%)	B/ACC(%)
			**14/99.30%**	22/99.30%	15/94.20%
Laboratory	412	40	**12/77.18%**	14/67.23%	26/72.09%

A: Number of original features; B: Number of selected features.

Table 11 shows the comparison with previous research on the open source dataset Yaseen. As can be seen from Table 11 that Yaseen got an accuracy of 97.9% by SVM in 2018 [25]. In 2020, Baghel achieved an accuracy of 98.6% using CNN [47]. In 2020, Oh attained an accuracy of 98.2% by WaveNet [48]. Our proposed method can achieve the best accuracy compared to the above algorithms.

**Table 11:** Comparison of results using Yaseen database.

Heart Sound Dataset	Authors	Year	Features	SVM(C)	DNN(C)	KNN(C)	RF(C)	DT(C)	CNN(C)	WaveNet(C)
Yaseen	Yaseen et al. [25]	2018	MFCCs and DWT	97.90%	92.10%	97.40%	/	/	/	/
Yaseen	Baghel et al. [47]	2020	/	/	/	/	/	/	98.60%	/
Yaseen	Oh et al. [48]	2020	/	/	/	/	/	/	/	98.20%
Yaseen	**Ours: EMD + Adaptive HD**	2022	Fusion features	/	/	**99.30%**	**99.30%**	94.20%	/	/

C: Accuracy(%).

## Conclusion

The heart sounds with valve defects were effectively distinguished from the normal heart sounds. This paper used adaptive Empirical Mode Decomposition (EMD) and feature fusion strategy to classify heart sounds. Several feature selection methods and classifiers were chosen to compare. Experimental tests in two databases validated the effectiveness of the proposed IMF reconstruction method with the adaptive Hausdorff Distance thresholds. Our proposed feature fusion technique with 40 features, including ordinary features and cardiac reserve time features, can achieve robust and excellent results. The experimental results show our proposed methods, adaptive EMD and feature fusion, are of great value to further realize the clinical auxiliary diagnosis of heart disease.

Although there is a good performance on the public dataset, the number and type of samples collected in our laboratory are insufficient. To achieve better experimental results, more samples need to be collected to verify the robustness and effectiveness of our proposed algorithm.

Some areas can still be optimized and improved to be studied in the future, such as obtaining more heart disease datasets, using ML and AI techniques to analyze heart sounds and improving the accuracy of heart sound classification, reducing the time-consuming cost of the algorithm.
